# Proteomics and acetyl-proteomics reveal the antibacterial mechanism of berberine sulfate against methicillin-resistant *Staphylococcus aureus*

**DOI:** 10.3389/fmicb.2025.1732962

**Published:** 2026-01-14

**Authors:** Zunli Hu, Huiling Liu, Mengying Chen, Jiafu Zhou, Yunxu Bian, Chuyao Lin, Shuming Liu, Yewen Sun, Minjia Tan, Jun-Yu Xu

**Affiliations:** 1 Guangzhou University of Chinese Medicine, Guangzhou, Guangdong, China; 2 Zhongshan Institute for Drug Discovery, Shanghai Institute of Materia Medica, Chinese Academy of Sciences, Guangdong, China; 3 School of Chinese Materia Medica, Nanjing University of Chinese Medicine, Nanjing, Jiangsu, China; 4 School of Pharmaceutical Sciences, Southern Medical University, Guangzhou, China; 5 State Key Laboratory of Drug Research, Shanghai Institute of Materia Medica, Chinese Academy of Sciences, Shanghai, China

**Keywords:** Berberine sulfate, lysine acetylation, methicillin-resistant *Staphylococcus aureus*, proteomic analysis, SarA

## Abstract

**Introduction:**

The rising prevalence of methicillin-resistant *Staphylococcus aureus* (MRSA) underscores an urgent need for novel antibacterial strategies. Although the natural antibacterial agent berberine sulfate exhibits inhibitory effects against MRSA, its precise molecular targets and mechanisms of action remain unclear.

**Methods:**

To elucidate its mechanism, this study employed quantitative proteomics to analyze protein expression changes in MRSA before and after drug treatment. Acetylomics was further applied to investigate the impact on post-translational modifications. Key findings were functionally validated using site-directed mutagenesis and electrophoretic mobility shift assays (EMSA).

**Results:**

Quantitative proteomics identified 255 differentially expressed proteins. Acetylomic analysis further revealed 38 differentially acetylated sites, among which berberine sulfate specifically induced acetylation at the K82 site of the global regulator SarA. Functional experiments demonstrated that the K82-mutated SarA protein showed significantly reduced binding affinity to the promoter of the virulence gene cluster *agr*.

**Discussion:**

These results indicate that berberine sulfate mediates a comprehensive stress response in MRSA through extensive alterations in protein expression and post-translational modifications. Specifically, the acetylation of SarA at K82 and the consequent impairment of its DNA-binding capacity represent a potential core mechanism by which berberine sulfate suppresses MRSA virulence and adaptability.

## 1 Introduction

*Staphylococcus aureus* (*S. aureus*) is a prevalent Gram-positive pathogen that colonizes approximately 20 to 41% of the human population and is associated with a wide range of infections, from minor skin abscesses to severe, life-threatening sepsis (Bhattacharya and Horswill, 2024). The excessive use of antibiotics has expedited the development of drug-resistant strains, notably methicillin-resistant *Staphylococcus aureus* (MRSA), which exhibits multidrug resistance and presents limited therapeutic options. This phenomenon has markedly increased morbidity and mortality rates in both human and animal populations (Idrees et al., 2021). MRSA can asymptomatically colonize the nares and skin or cause mild skin and soft tissue infections. However, it can also result in severe invasive diseases, including bacteremia, endocarditis, deep surgical site infections, and necrotizing pneumonia (Flynn and Guarner, 2023). Current treatment strategies primarily rely on last-resort antibiotics such as vancomycin and linezolid. Nevertheless, the emergence of vancomycin-intermediate (VISA) and vancomycin-resistant *Staphylococcus aureus* (VRSA) strains has further constrained therapeutic options (Fair and Tor, 2014). This crisis highlights the urgent need to develop novel strategies to combat MRSA.

Natural products have emerged as invaluable resources in combating antibiotic resistance, attributed to their structural diversity and unique bioactive mechanisms. In the development of therapeutics targeting *S. aureus*, particularly MRSA, natural products demonstrate exceptional potential. Notably, approximately 40% of clinically utilized antimicrobial agents are derived, either directly or indirectly, from natural sources (Newman and Cragg, 2020), highlighting their critical role in drug discovery. Various natural products exhibit significant anti-MRSA activity, including plant-derived compounds such as garlic extracts and microbial secondary metabolites like streptomycete antibiotics (Chen et al., 2020; Liu et al., 2022). Among these, plant secondary metabolites are especially appealing due to their structural complexity and low toxicity to host organisms.

Berberine sulfate, an isoquinoline alkaloid predominantly found in Coptis species, has been shown to possess a diverse array of pharmacological properties, including anti-inflammatory, antioxidant, hypocholesterolemic, antidiabetic, and anti-obesity effects (Kong et al., 2004; Kumar et al., 2015; Wang et al., 2017; Neag et al., 2018). Notably, it also exhibits potent antimicrobial activity against *Escherichia coli* (Li and Ge, 2023), *Streptococcus agalactiae* (Peng et al., 2015), and MRSA (Zhou et al., 2024). Nevertheless, the current comprehension of berberine’s anti-MRSA activity is largely confined to its synergistic interactions with antibiotics such as clindamycin and rifamycin, as well as its mechanisms involving membrane and cell wall disruption (Xia et al., 2022). The complex molecular pharmacology of berberine sulfate against MRSA remains inadequately understood, particularly regarding its proteomic and post-translational regulatory networks.

Contemporary advancements in mass spectrometry-based quantitative proteomics have established it as an essential instrument for unraveling the antimicrobial mechanisms of small molecules, offering comprehensive molecular insights into protein expression and post-translational modifications. Prior research has effectively utilized this technology to elucidate the anti-MRSA mechanisms of various natural products, such as cinnamaldehyde (Chen et al., 2024), flavonoids (Weng et al., 2024), and theaflavins (Guan et al., 2022). Our research group has further expanded this methodology to demonstrate how quercetin and caffeic acid influence the metabolic network of mycobacteria (Zhang et al., 2022). Building upon these foundational studies, the present investigation will, for the first time, integrate quantitative proteomics with acetylome profiling to systematically examine the alterations induced by berberine sulfate in both the proteome and post-translational modification networks of MRSA.

In this study, we utilized quantitative proteomics to examine the proteomic alterations in MRSA subsequent to treatment with berberine sulfate. Our analysis identified a total of 1,685 proteins, among which 111 were significantly upregulated and 144 were downregulated. Functional enrichment analysis indicated that the differentially expressed proteins were predominantly associated with cytoplasmic metabolic processes. Notably, our acetylome profiling revealed, for the first time, a significant acetylation modification at lysine 82 (K82) of the global regulator SarA. Subsequent validation analyses demonstrated that the SarA mutant exhibited impaired DNA-binding capacity in comparison to its wild-type counterpart. These findings not only elucidate the mechanism by which berberine sulfate modulates virulence factors through acetylation, but also propose novel strategies for the design of anti-MRSA therapeutics targeting virulence regulation. Specifically, the regulatory enzymes responsible for the acetylation of SarA at K82 might be potential therapeutic targets.

## 2 Materials and methods

### 2.1 Determination of the minimum inhibitory concentration of berberine sulfate against *Staphylococcus aureus*

The minimum inhibitory concentration (MIC) of berberine sulfate (Felix, Shanghai, China; CAS: 633–66-9) against methicillin-resistant *Staphylococcus aureus* (MRSA) strain ATCC 33591 was determined using the microbroth dilution method (Humphries et al., 2021). Briefly, 90 μL of serially twofold-diluted berberine sulfate solution was dispensed into each well of a 96-well plate to achieve final concentrations ranging from 2 to 256 μg/mL. Vancomycin was included as a positive-control antibiotic. A bacterial suspension was adjusted to a 0.5 McFarland standard and inoculated at a 1:1000 ratio into each well. All experiments were performed with three independent biological replicates (*n* = 3). The MIC was defined as the lowest concentration of the agent that completely inhibited visible growth after incubation.

In all functional experiments in this study, drug concentrations were expressed in micromolar (μM). The molecular weight of berberine sulfate was 433.43 g/mol (calculated based on the chemical formula C_20_H_18_NO_8_·SO4). The drug concentrations used in the experiments were obtained by proportional conversion based on the minimum inhibitory concentration (MIC) measured via the microbroth dilution method. The conversion between mass concentration (μg/mL) and molar concentration (μM) was performed according to the following formula:


$$\begin{array}{l} \text{Concentration}(\mathrm{\mu M})=\\ {}[\text{Concentration}(\mathrm{\mu g}/\mathrm{mL})/433.43\;g/\mathrm{mol}]\times 1000 \end{array}$$

The experimental concentrations reported in the text were all micromolar concentrations converted based on the above formula. Unless otherwise specified, concentrations mentioned in the text refer to μM, except for the raw MIC values reported in the “Results” section.

### 2.2 Growth curve assay

The growth inhibitory effect of berberine sulfate was assessed using a 96-well microplate broth dilution method. Briefly, serially diluted berberine sulfate solutions were prepared, and 10 μL of each dilution was added to individual wells to achieve final concentrations corresponding to the MIC, 1/2 MIC, and 1/6 MIC in a total volume of 100 μL per well. Control wells containing only culture medium without the drug were included. All conditions were tested in triplicate. Subsequently, a freshly prepared bacterial suspension was adjusted to a 0.5 McFarland standard using Mueller-Hinton Broth (MHB, Haibo, and HB6231) and then diluted 1:1000 before being added to each well. The plate was incubated statically at 37 °C. Bacterial growth was monitored by measuring the optical density at 600 nm using a microplate reader at selected time points (0, 2, 4, 6, 8, 12, 14, 18, and 24 h). Growth curves were plotted with incubation time on the x-axis and the mean OD₆₀₀ values on the y-axis to systematically analyze the growth kinetics under different drug concentrations.

### 2.3 Time-kill curve assay

To evaluate the bactericidal activity of berberine sulfate against MRSA, we conducted a time-kill kinetics study following previously reported procedures (Blaskovich et al., 2018). A freshly prepared bacterial culture was adjusted to a 0.5 McFarland standard in MHB and subsequently diluted 1:1000 for inoculation into each well. This diluted suspension was dispensed into 96-well microplates and combined with 10 μL of berberine sulfate solution at a concentration equivalent to 1/6 of the MIC, resulting in a final reaction volume of 100 μL per well. The plates were then incubated statically at 37 °C.

At selected time points (0, 4, 8, 12, and 16 h), aliquots were withdrawn from each well, serially diluted as appropriate, and spread onto Mueller-Hinton Agar (MHA, Haibo, HB6231) plates. After overnight incubation at 37 °C, visible colonies were enumerated. The mean colony counts, expressed as log₁₀ CFU/mL after accounting for dilution factors, were plotted against time to generate time-kill curves. These curves were used to analyze the dynamic effects of sub-inhibitory concentrations of berberine sulfate on MRSA viability.

### 2.4 Preparation of proteomic samples

To prepare samples for proteomic analysis, an overnight culture of MRSA was inoculated into MHB medium and incubated with shaking at 37 °C until reaching the mid-logarithmic growth phase (OD₆₀₀ = 0.2). Berberine sulfate was then added to the culture to a final concentration of 50 μM, a sub-inhibitory concentration, followed by further incubation for 24 h. This entire treatment process was performed in duplicate as two independent biological replicates. After the incubation, the bacterial cells were harvested by centrifugation at 9,289 × g for 5 min at 4 °C. The supernatant was discarded, and the resulting cell pellet was washed twice with ice-cold phosphate-buffered saline (PBS).

The collected bacterial pellet was resuspended in lysis buffer (8 M urea, 100 mM NH_4_HCO_3_, pH 8.0) containing 1 × protease inhibitor, 2 × phosphatase inhibitor, 10 mM NaF, 1 mM PMSF, and phosphatase inhibitor cocktails 2 and 3. The suspension was incubated on ice for 30 min, followed by sonication for 7 min. Subsequently, the lysate was centrifuged at 21,300 × g for 10 min at 4 °C, and the supernatant was collected. Protein concentration was determined using the bicinchoninic acid (BCA) protein assay kit (Beyotime, P0011).

### 2.5 In solution tryptic digestion

The protein extract was reduced with a final concentration of 5 mM dithiothreitol at 56 °C for 30 min, followed by alkylation with a final concentration of 15 mM iodoacetamide in the dark at 25 °C for 30 min. After the reaction, a final concentration of 30 mM L-cysteine was added and allowed to react at 25 °C for 30 min. The lysate was then diluted with 100 mM NH₄HCO₃ to reduce the final urea concentration to 2 M. Trypsin digestion was conducted at 37 °C for 16 h with a trypsin-to-protein ratio of 1:50 (*w*/*w*). Subsequently, LysC was added at a LysC-to-protein ratio of 1:100 (*w*/*w*), and the mixture was incubated at 37 °C for 4 h. The peptide samples were desalted using a Sep-Pak Vac C18 column and eluted with 75% acetonitrile (ACN) containing 0.1% trifluoroacetic acid (TFA). Finally, peptides samples were dried using a SpeedVac (SPD120, Thermo Fisher Scientific, Waltham, MA, United States).

### 2.6 TMT labeling

The 360 μg of desalted peptides were dissolved in 720 μL of a 100 mM TEAB solution, with the pH adjusted to 8.0. For efficient labeling, the peptides were treated with TMT 6-plex Isobaric Label Reagent (90,066, Thermo, United States) reagent dissolved in acetonitrile (ACN) at a peptide-to-TMT reagent ratio of 1:2. The TMT labeling channels 126 to 129 N were assigned as follows: 126: control 1; 127 N: control 2; 128C: berberine sulfate 1; 129 N: berberine sulfate 2. After incubation at 25 °C for 1 h, the reaction was quenched with 5% hydroxylamine. The labeling efficiency was confirmed to be satisfactory, exceeding 95%. Subsequently, the peptides labelled by different tags were mixed were desalted and dried.

### 2.7 Affinity enrichment of acetylated lysine peptides

Peptides were dissolved in 450 μL of ETN buffer (50 mM Tris–HCl, 1 mM EDTA-2Na, 600 mM NaCl, adjusted to pH 8.0 with 5 M NaOH and 1 M HCl). The samples were then loaded onto agarose beads containing acetylated lysine antibodies (Immune Chem, catalog number ICP0380). After thorough mixing, all samples were incubated with shaking overnight at 4 °C. The following day, the supernatant was collected, and the beads were washed with NETN buffer (1 mM EDTA-2Na, 50 mM Tris–HCl, 0.5% NP-40, 600 mM NaCl, pH 8.0), ETN, and double-distilled water. Finally, the peptides were eluted with mass spectrometry grade water which contained 30% acetonitrile and 0.1% TFA. The concentrated acetylated peptides were desalted and dried using a ZipTip C18 column (Millipore, ZTC18S960) in preparation for subsequent LC–MS/MS analysis.

### 2.8 LC–MS/MS analysis

The dried peptides were dissolved in Buffer A (100% H_2_O, 0.1% formic acid) and loaded onto a DNV75150PN-C18 column (Thermo Fisher Scientific, 15 cm length × 75 μm inner diameter, 2 μm particle size). The samples were analyzed using an Vanquish Neo UHPLC (Thermo Fisher Scientific) coupled with an Orbitrap Ascend mass spectrometer (Thermo Fisher Scientific). The peptides were eluted using buffer B (80% acetonitrile, 20% H_2_O, 0.1% formic acid). For the proteome profiling samples, the MS data acquisition was performed in data-independent acquisition (DIA) mode using Xcalibur software (version 4.6). The elution gradient was set to 18 min with an initial flow rate of 700 nL/min (0–1.8 min, 4–4.5% buffer B), followed by a flow rate of 300 nL/min (2–14 min, 6–35% buffer B), then followed by a flow rate of 700 nL/min (14–14.5 min, 35–55% buffer B; 14.5–15 min, 99% buffer B; 15–18 min, 99% buffer B). The MS1 full scans were acquired at a resolution of 120,000. MS data acquisition was performed in data-independent acquisition (DIA) mode using 60 variable windows covering a mass range of 380–980 m/z. The automatic gain control (AGC) target was set to 8.0 × 10^5^, with a maximum injection time (IT) of 10 ms. The MS2 scans were performed over a mass range of m/z 150 to 2,000 at a resolution of 30,000. The AGC target for MS2 was 1 × 10^5^, with a maximum IT of 59 ms. The precursor ions were fragmented using high-energy collision dissociation (HCD) mode with a normalized collision energy set at 25%.

For TMT-labeled peptides samples, the elution gradient was set to 45 min with an initial flow rate of 500 nL/min (0–0.5 min, 5–7% buffer B), followed by a flow rate of 300 nL/min (0.5–33 min, 7–30% buffer B), then followed by a flow rate of 500 nL/min (33–38 min, 30–50% buffer B; 38–41 min, 99% buffer B; 41–45 min, 99% buffer B). The eluted peptides were subjected to MS1 full-scan analysis in the Orbitrap at a resolution of 120,000 with a scan range set at 400–1,600 m/z. The automatic gain control (AGC) was 8.0 × 10^5^ and the maximum ion injection time (IT) was 50 ms. For MS2, high-energy collision dissociation (HCD) fragmentation was used, and parent ions and precursors with charge states of 2 to 6 were fragmented using 35% HCD collision energy and then detected in the Orbitrap at a resolution of 15,000, with a scan range set at 110–2,000 m/z. The AGC was 1.0 × 10^5^ and the maximum injection time was 27 ms.

For TMT-labeled acetylated peptides samples, the elution gradient was set to 110 min with an initial flow rate of 500 nL/min (0–0.5 min, 5–7% buffer B), followed by a flow rate of 300 nL/min (0.5–98 min, 7–30% buffer B), then followed by a flow rate of 500 nL/min (98–103 min, 30–50% buffer B; 103–106 min, 99% buffer B; 106–110 min, 99% buffer B). The MS parameters were set the same as those for TMT-labeled peptide samples, except for the maximum injection time for MS2, which was set to 200 s. Moreover, dynamic exclusion was set to 30 s.

### 2.9 MS database searching

The LC–MS/MS raw data were analyzed using DIA-NN software (version 1.8.1) and MaxQuant software (version 2.4.14.0) against the UniProt *S. aureus* database (2,889 proteins, Proteome ID: UP000008816, updated on 03/13/2024). Trypsin/P was specified as the enzyme, allowing for up to two missed cleavages. The false discovery rate (FDR) for proteins, peptides, and sites was set at 1%. For the proteomics data, carbamidomethylation (C) was designated as a fixed modification, while acetylation (Protein N-term) and oxidation (M) were designated as variable modifications. MS2 reporter ions were quantified using TMT correction factors. For the acetylome data, acetylation (K) was also included as a variable modification. Other fixed and variable modifications were consistent with the proteomics analysis. Acetylation sites with localization probabilities greater than 0.75 were selected for further bioinformatics analysis.

### 2.10 Differential protein and acetylation site analyses

For the differential protein analysis, proteins identified solely by site were excluded, along with reverse or potential contaminant proteins. To ensure data comparability, the intensity of each channel was normalized to the median of the corresponding channel, followed by log2 transformation. Student’s t-test was employed to evaluate the differences in protein expression levels between the control and berberine sulfate groups, yielding *p*-values. The average channel intensities for both the control and berberine sulfate groups were then calculated, and fold-change (FC) values were determined by comparing the berberine sulfate group to the control group. Proteins with *p* < 0.05 and FC > 1.5 were considered upregulated, while those with *p* < 0.05 and FC < 0.67 were deemed downregulated. For the differential acetylation site analysis, reverse or potential contaminant proteins, as well as acetylation sites with localization probabilities < 0.75, were excluded. The intensity of each channel was normalized using the protein expression profile data, followed by log2 transformation, with the remaining data processed in the same manner as for the differential protein analysis. Student’s t-test was utilized to assess the differences in acetylation levels between the control and berberine sulfate groups. Acetylation sites with *p* < 0.05 and FC > 1.2 were classified as upregulated, while those with *p* < 0.05 and FC < 0.83 were classified as downregulated.

### 2.11 Bioinformatics analysis

Bioinformatics analysis excluded reverse and contaminating proteins, retaining only modified proteins with a localization probability greater than 0.75. Two-tailed t-tests were employed to assess significant differences in protein expression levels. Pathway enrichment analysis, keyword annotation, and the construction of protein–protein interaction (PPI) networks were conducted using the STRING database (version 12.0). Interaction networks with medium confidence scores of 4 or higher were visualized using Cytoscape (version 3.10.1), and highly enriched clusters were analyzed with the MCODE plugin.

### 2.12 Plasmid construction of wild-type and mutant genes

Primers for amplification, site-directed mutagenesis, and sequencing were designed using the professional software SnapGene® (version 6.0.2). To construct the wild-type plasmid, the bacterial genomic DNA extraction kit (TIANGEN, DP302-02) was utilized to extract genomic DNA from *S. aureus.* The wild-type target genes in *S. aureus* DNA were amplified using Phanta® Max Super-Fidelity DNA Polymerase (Vazyme, P515) along with the appropriate primers. The PCR products were purified using the Universal DNA Purification and Recovery Kit (TIANGEN, DP214-03). Subsequently, the purified PCR products were ligated into a pET28a (+) expression vector.

For the construction of the mutant plasmid, the wild-type recombinant plasmid served as the template DNA. The sequence of the target mutant gene was amplified using the KOD Plus high-fidelity PCR enzyme (TOYOBO, KOD-401) in conjunction with primers designed for site-directed mutagenesis. Two reaction mixtures were prepared, each containing 100 ng of the wild-type plasmid as the PCR template. After completing the PCR reaction, the products from both tubes were combined into a single tube, and an additional 1 μL of KOD enzyme was added. The PCR reaction was then repeated. Subsequently, 1 μL of FastDigest DpnI (Thermo Fisher Scientific, FD1704) was added, and the reaction was incubated at 37 °C for 1 h to digest the template. The PCR product was purified using a Universal DNA Purification and Recovery Kit (TIANGEN, DP214-03) to isolate the site-directed mutant plasmid.

The wild-type and mutant recombinant plasmids were transformed into DH5α chemically competent cells (Tsingke, TSC-C14) and subsequently sequenced. Finally, the recombinant plasmids with confirmed correct sequencing results were transformed into BL21 (DE3) cells (Tsingke, TSCE06).

### 2.13 Inducible expression and purification of the target protein

The wild-type and mutant strains of the target protein were inoculated into LB medium supplemented with 50 μg/mL kanamycin and cultured at 37 °C with shaking at 220 rpm for 16 h. When the optical density (OD_600_) reached between 0.4 and 0.6, 0.1 mM isopropyl-*β*-D-thiogalactoside (IPTG) was added to induce protein expression. The cultures were then incubated at 16 °C with shaking at 160 rpm for an additional 16 h. After induction, the bacterial cells were harvested by centrifugation at 9,289 × g for 5 min at 4 °C, and the supernatant was discarded. The bacterial pellet was resuspended in 10 mM imidazole buffer (PBS) and mixed thoroughly to ensure complete resuspension. The resuspended cells were sonicated for 5 min and then centrifuged at 10,414 × g for 20 min. The supernatant of proteins was collected, and the target proteins were purified using Ni-NTA agarose resin columns.

### 2.14 *In vitro* DNA-binding activity assays of SarA

In this section, we employed the Electrophoretic Mobility Shift Assay (EMSA) to investigate the interactions between proteins and nucleic acids. The detailed experimental procedures were performed according to reference (Stowell et al., 2021). After purifying the proteins, the samples were mixed with EMSA/Gel-Shift loading buffer and sterile water, then incubated at 18 °C for 10 min. Subsequently, nucleic acid probes were added, and the mixture was incubated for an additional 20 min. Before loading, 1 μL of EMSA/Gel-Shift loading buffer (Beyotime, GS009-2) was added to the 10 μL reaction mixture. The sample mixture was carefully loaded into the wells of the EMSA gel, followed by electrophoresis at a constant voltage of 80 V under cold conditions (4 °C). After electrophoresis, the probes, protein-nucleic acid complexes, and free nucleic acids were transferred to a nylon membrane. The membrane was then exposed to UV light for 15 min to cross-link the transferred molecules. Following cross-linking, the membrane was incubated with blocking buffer (Beyotime, GS009-7) and subsequently with blocking buffer containing the antibody (Streptavidin-HRP Conjugate; Beyotime, GS009-6) on a side-to-side shaker for 15 min. The membrane was then washed with washing buffer (Beyotime, GS009-8). Finally, the membrane was incubated with a mixture of BeyoECL Moon working solutions A and B (GS009-4, GS009-5) at room temperature for 3 min and imaged in chemiluminescence mode using a fluorescence and chemiluminescence imaging system (Clinx, ChemiScope 6,200).

### 2.15 Statistical analysis

The statistical analyses were conducted using Student’s t-test with GraphPad Prism version 10.1.2. The data were expressed as the mean ± standard deviation. Values of *p* < 0.05 were considered statistically significant.

## 3 Results

### 3.1 Inhibitory effects of berberine sulfate on MRSA

**Figure 1:**
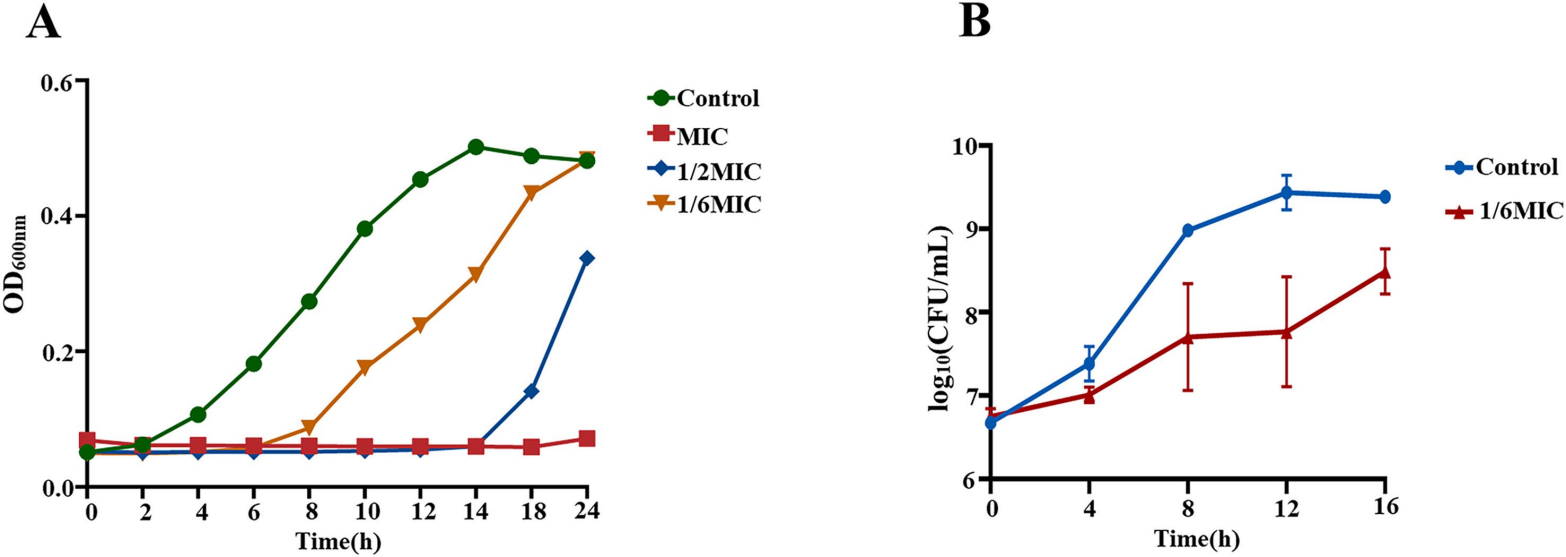
Growth inhibition and time-kill kinetics of berberine sulfate against MRSA. **(A)** Growth curves of MRSA treated with different concentrations of berberine sulfate; **(B)** Time-kill curve of MRSA exposed to 1/6 MIC berberine sulfate. Data points represent the mean ± standard deviation of three independent experiments.

The minimum inhibitory concentration (MIC) of berberine sulfate against MRSA was determined to be 295.3 μM using the microbroth dilution method. Growth curve analysis revealed a concentration-dependent inhibitory effect of berberine sulfate on MRSA. At the MIC, bacterial growth was completely suppressed over the 24 h incubation period. Sub-inhibitory concentrations also significantly altered bacterial growth dynamics: at 1/2 MIC, proliferation was noticeably delayed, and at 1/6 MIC, the growth rate was markedly reduced (Figure 1A). This trend was further corroborated by the time-kill assay, in which the viable bacterial counts in cultures treated with 1/6 MIC berberine sulfate were significantly lower than those in the untreated control at all time points (Figure 1B). Together, these results demonstrate that even at sub-inhibitory concentrations, berberine sulfate can persistently inhibit the proliferation and survival of MRSA. Subsequently, biofilm formation assays were conducted. The results indicated that while berberine sulfate exhibited significant bactericidal activity at the tested concentrations, it did not lead to a statistically significant reduction in biofilm biomass (Supplementary Figure S1).

**Figure 2:**
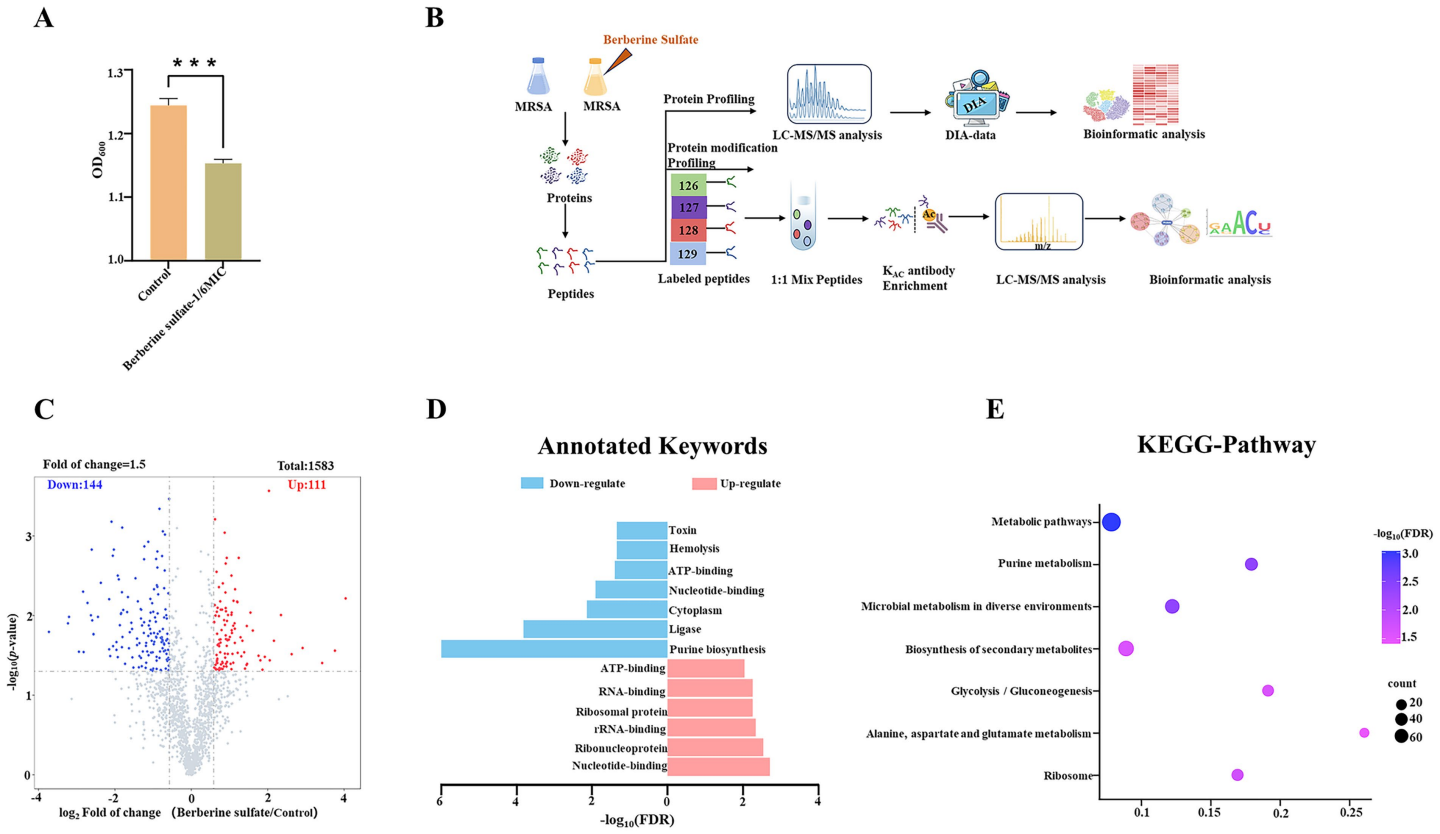
Proteomic experimental design and analysis to investigate the mechanism of berberine sulfate inhibition against MRSA. **(A)** Analysis of the antibacterial activity of berberine sulfate (50 μM, 24 h treatment) against MRSA (*n* = 2); **(B)** Experimental design for the proteomic analysis of berberine sulfate intervention in MRSA using a TMT labeling strategy. **(C)** Volcano plot illustrating the proteins that exhibit statistically significant changes in the berberine sulfate-treated group; **(D)** Keyword annotations for the up- and down-regulated proteins in the proteomic expression profiles; **(E)** KEGG pathway analysis of the up- and down-regulated proteins.

To further investigate its antibacterial mechanism, we first assessed the effect of a sub-inhibitory concentration (50 μM, approximately 1/6 MIC) of berberine sulfate on subsequent bacterial growth when MRSA cultures reached an OD_600_ of 0.2. As shown in Figure 2A, bacterial proliferation was significantly inhibited at this concentration.

To elucidate the molecular mechanism of berberine sulfate, we treated MRSA with a subinhibitory concentration (50 μM) of the compound and performed quantitative proteomic analysis (Figure 2B). Global proteomic profiling using data-independent acquisition (DIA) identified a total of 1,685 proteins. Compared with the control group, the treatment group showed significant changes in the expression of 255 proteins (screening criteria: *p* < 0.05, fold change ≥ 1.5), including 111 upregulated and 144 downregulated proteins (Figure 2C; Supplementary Table S1).

### 3.2 Proteomic profiling of MRSA in response to berberine sulfate treatment

**Figure 3:**
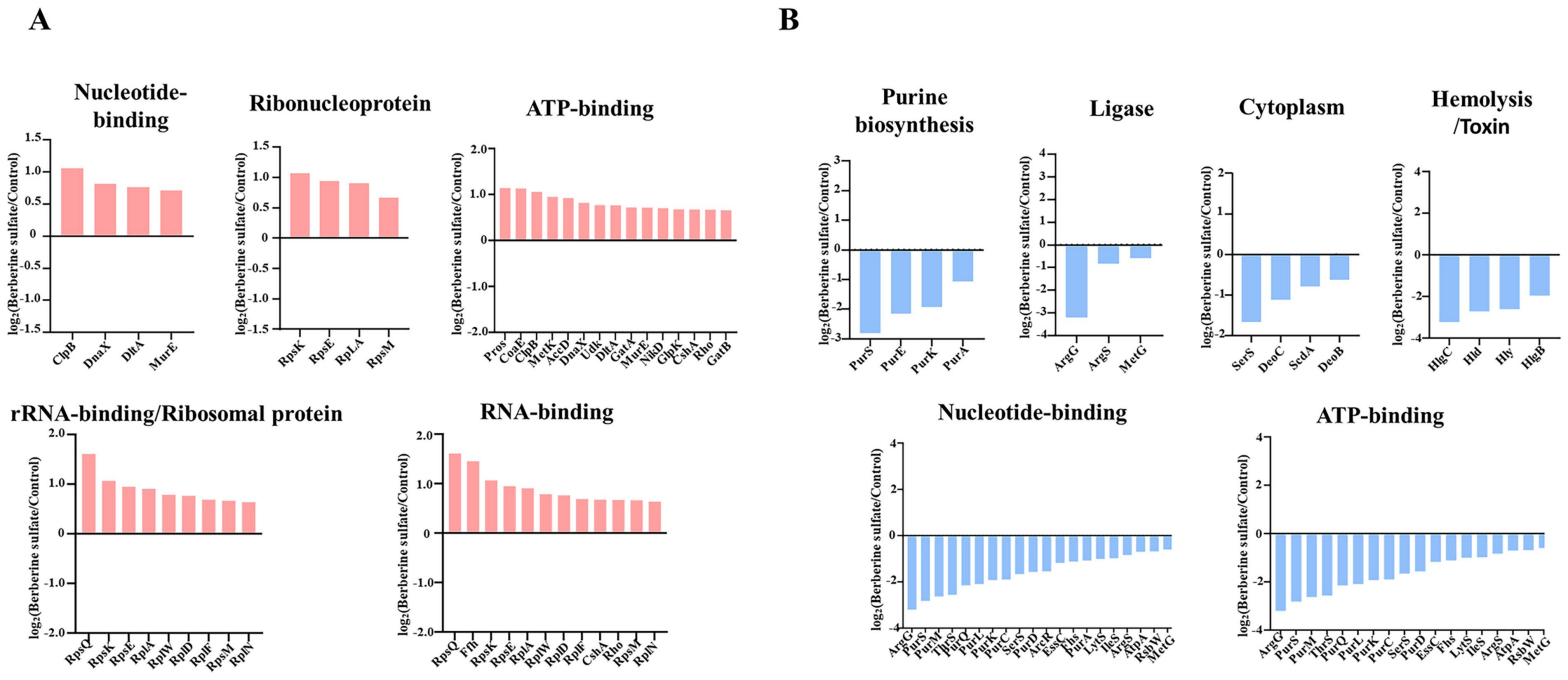
Landscape of key protein pathways in MRSA in response to berberine sulfate. **(A)** Bar plot showing the identified upregulated proteins; **(B)** Bar plot showing the identified downregulated proteins.

**Figure 4:**
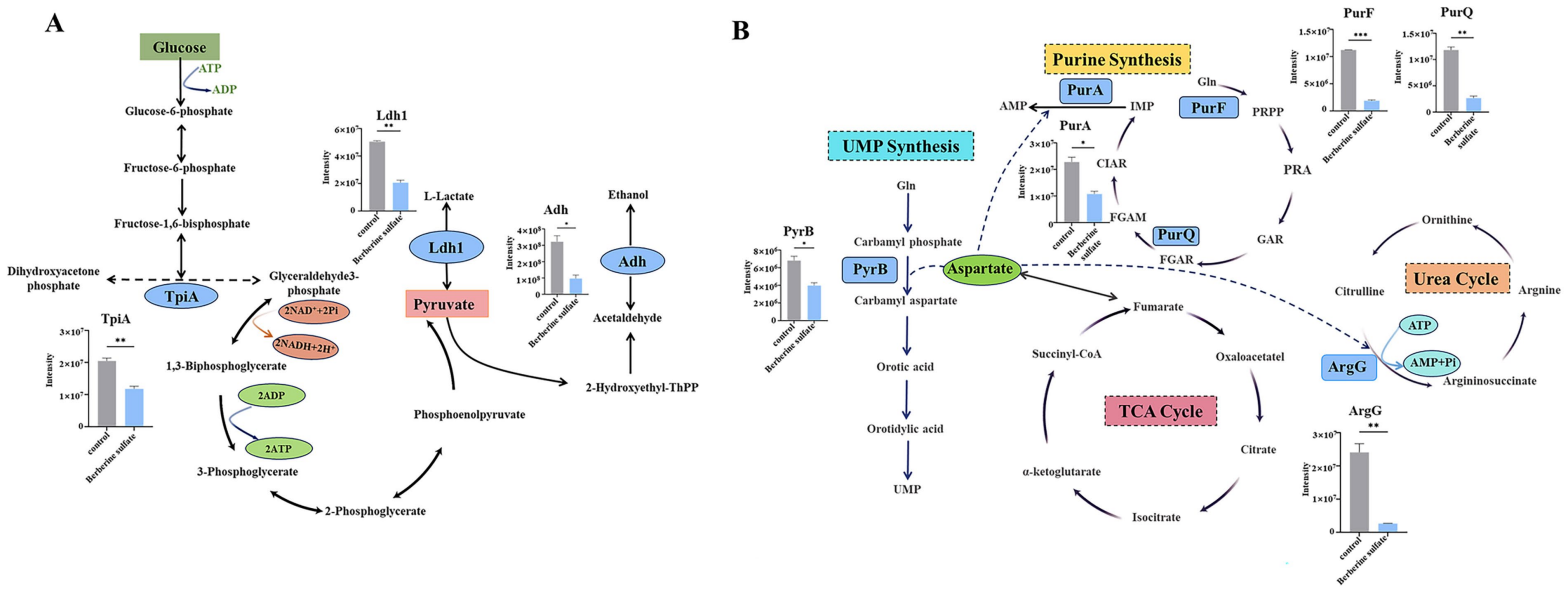
Key metabolic pathways and associated protein expression. **(A)** Glycolysis pathway annotated with identified enzymes: TpiA, triosephosphate isomerase; Ldh1, L-lactate dehydrogenase 1; Adh, alcohol dehydrogenase; **(B)** the alanine, aspartate, and glutamate metabolism pathway annotated with identified enzymes: PyrB, aspartate carbamoyltransferase catalytic subunit; PurA, adenylosuccinate synthetase; PurQ, phosphoribosylformylglycinamidine synthase subunit PurQ; PurF, amidophosphoribosyltransferase; ArgG, argininosuccinate synthase.

**Figure 5:**
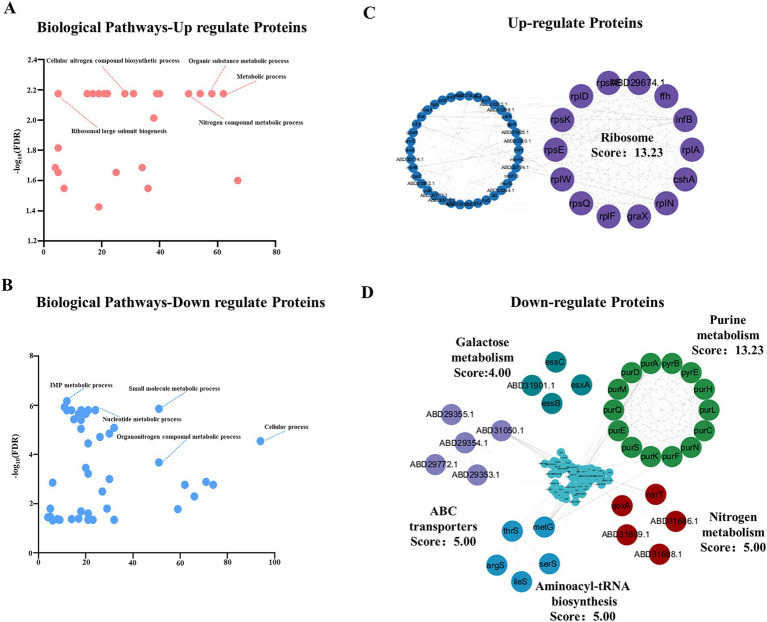
Biological pathway and protein interaction network analysis of MRSA differential proteins treated with berberine sulfate. **(A,B)** Pathway analysis of upregulated **(A)** and downregulated **(B)** proteins in MRSA treated with berberine sulfate; **(C,D)** protein–protein interaction networks of upregulated proteins **(C)** and downregulated proteins **(D)** in the berberine sulfate-treated group.

To further elucidate the biological significance of the differentially expressed proteins in MRSA following berberine sulfate treatment, a systematic bioinformatic analysis was performed. Analysis of annotated keywords indicated that these differentially expressed proteins were predominantly localized in the cytoplasm (Figure 2D). KEGG pathway analysis revealed significant enrichment in metabolic pathways, notably within the “Biosynthesis of secondary metabolites” category (Figure 2E). In-depth analysis of the enriched pathways revealed that the compound employs a dual regulatory strategy to modulate core cytoplasmic processes: on one hand, it induced a comprehensive upregulation of ribosomal functional categories (including ribosomal proteins, rRNA-binding proteins, and ribonucleoproteins), manifested by a global increase in nearly all associated enzymatic components (Figure 3A); on the other hand, it coordinately suppressed key cytoplasmic enzymes involved in *de novo* purine synthesis (e.g., PurS, PurL) and the protein translation machinery (e.g., IleS, MetG, and SerS) (Figure 3B). This coordinated downregulation uncovered a dual-target inhibitory mechanism that concurrently disrupts nucleotide biosynthesis and protein synthesis—two fundamental biological processes—thereby compromising the biosynthetic capacity of MRSA. Furthermore, specific perturbations in metabolic pathways were equally prominent: the expression levels of three key glycolytic enzymes (TpiA, Ldh1, and Adh) were significantly reduced, while multiple catalytic enzymes (PyrB, PurA, PurQ, PurF, and ArgG) in the alanine, aspartate, and glutamate metabolic pathways also exhibited consistent abundance decreases (Figures 4A,B). Further analysis of biological pathways demonstrated that berberine sulfate treatment resulted in the upregulation of essential metabolic processes, including general metabolic processes and organic substance metabolic processes, while downregulating small molecule metabolic processes and associated pathways (Figures 5A,B). These findings suggest that berberine sulfate exerts its antibacterial effects through the modulation of complex biological functions, thereby elucidating its potential pharmacological mechanisms and therapeutic targets.

### 3.3 Protein network analysis of the proteins with changed expression level in response to berberine sulfate treatment

To systematically elucidate the functional relationships among these differentially expressed proteins, we constructed a protein–protein interaction (PPI) network utilizing the STRING database and conducted modular analysis with the MCODE plugin in Cytoscape (score > 4). Notably, four highly interactive clusters were identified among the upregulated proteins. The cluster with the highest score (13.23) predominantly consisted of ribosomal proteins (Figure 5C). In contrast, the downregulated proteins were primarily enriched in energy metabolism pathways, including purine metabolism (score:13.23) and nitrogen metabolism (score:5.00) (Figure 5D). These interaction modules collectively suggest that berberine sulfate may exert its antibacterial effects through the coordinated regulation of multiple metabolic pathways in MRSA, encompassing energy metabolism and nucleotide biosynthesis.

### 3.4 Alterations in acetylation levels of MRSA following berberine sulfate intervention

**Figure 6:**
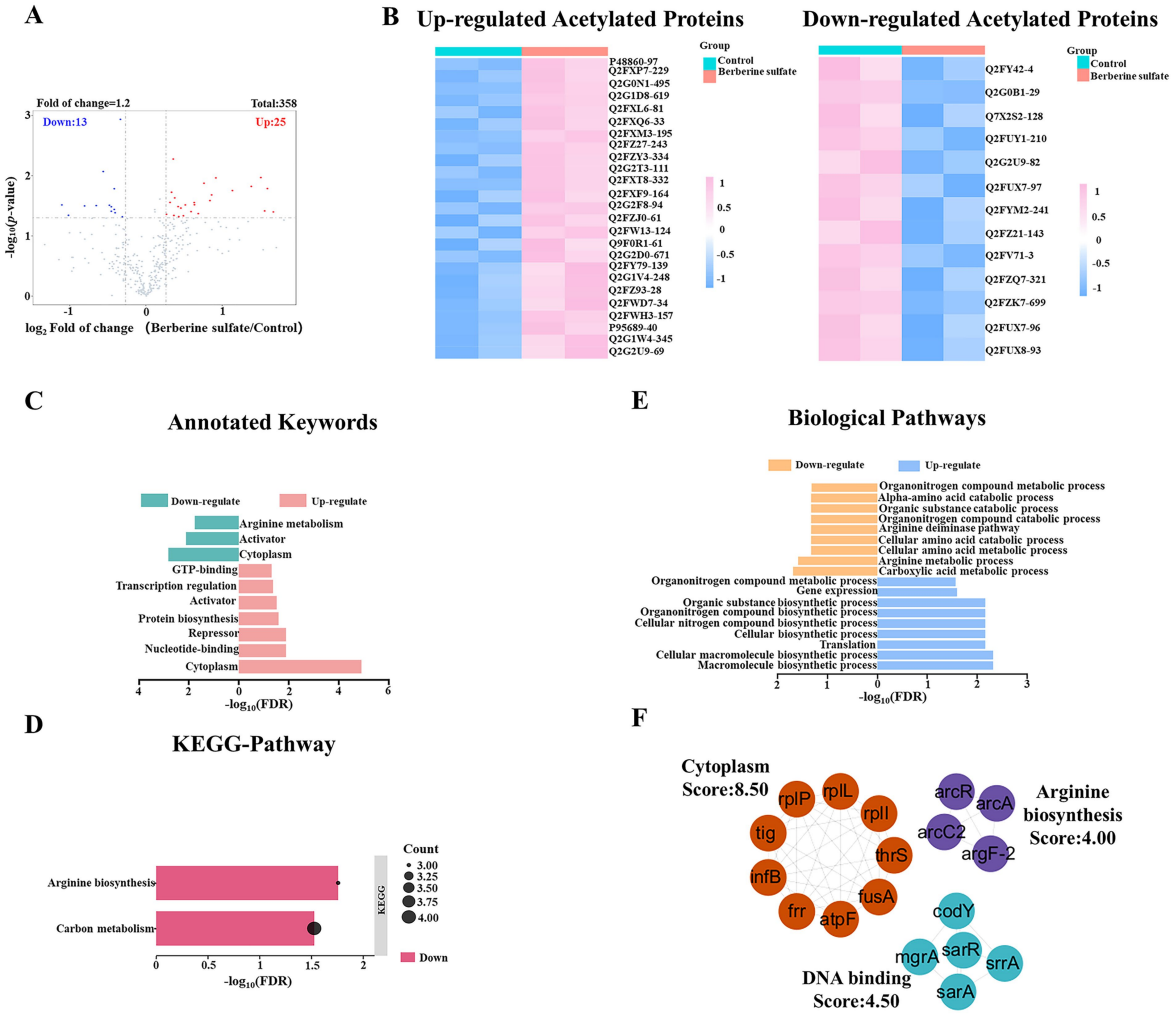
Overview of acetylation level changes in MRSA treated with berberine sulfate. **(A)** Dynamic landscape of acetylation modification sites in MRSA treated with berberine sulfate; **(B)** Heatmap showing identified acetylation sites that were upregulated or downregulated in MRSA in response to berberine sulfate treatment; **(C)** Functional annotation of upregulated and downregulated acetylation sites; **(D)** Downregulated acetylation modification characteristics identified through functional annotation of the KEGG pathway; **(E)** Biological pathways enriched with upregulated and downregulated acetylation sites; **(F)** The protein–protein interaction network of lysine-acetylated proteins.

This study examined the dynamic alterations in protein acetylation within MRSA subsequent to berberine sulfate intervention, identifying a total of 715 acetylation sites, of which 25 were significantly upregulated and 13 were downregulated (Figure 6A; Supplementary Tables S2, S3A,B). Heatmap analysis further revealed a global reprogramming pattern of acetylation sites, showing distinct clusters of up- and down-regulated sites (Figure 6B). Functional annotation of these differentially acetylated sites indicated a significant enrichment of upregulated sites in cytoplasmic localization, activator activity, and nucleotide-binding functions. Conversely, downregulated sites were predominantly associated with cytoplasmic proteins and pathways related to arginine metabolism (Figure 6C). It is noteworthy that cytoplasmic localization was significantly enriched among both upregulated and downregulated acetylation sites, implying that berberine sulfate may influence its biological effects through the modulation of protein acetylation states within the cytoplasmic compartment. Additionally, KEGG pathway analysis revealed a downregulation of acetylation modifications in pathways involved in arginine biosynthesis and carbon metabolism (Figure 6D).

At the biological process level, the differentially acetylated sites were predominantly associated with essential processes, such as macromolecule biosynthesis, carboxylic acid metabolism, and cellular macromolecule biosynthesis (Figure 6E). These observations suggest that berberine sulfate exerts its antibacterial effects by specifically modulating the protein acetylation network in MRSA, particularly through alterations in the acetylation states of cytoplasmic metabolic proteins. This modulation disrupts key metabolic pathways and biosynthetic processes. The extensive regulation of post-translational modification networks likely constitutes a fundamental molecular mechanism underlying the antimicrobial activity of berberine sulfate. To investigate functional interactions, a protein–protein interaction network was constructed using the STRING database, and three highly interconnected clusters were identified via the MCODE plugin in Cytoscape (Figure 6F). Notably, cluster 1 (score:8.50) demonstrated significant functional enrichment for cytoplasmic processes, corroborating the results of the keyword analysis. This consistency further supports the hypothesis that berberine sulfate modulates cytoplasmic acetylation.

### 3.5 Lysine acetylation inhibiting DNA binding affinity of SarA

**Figure 7:**
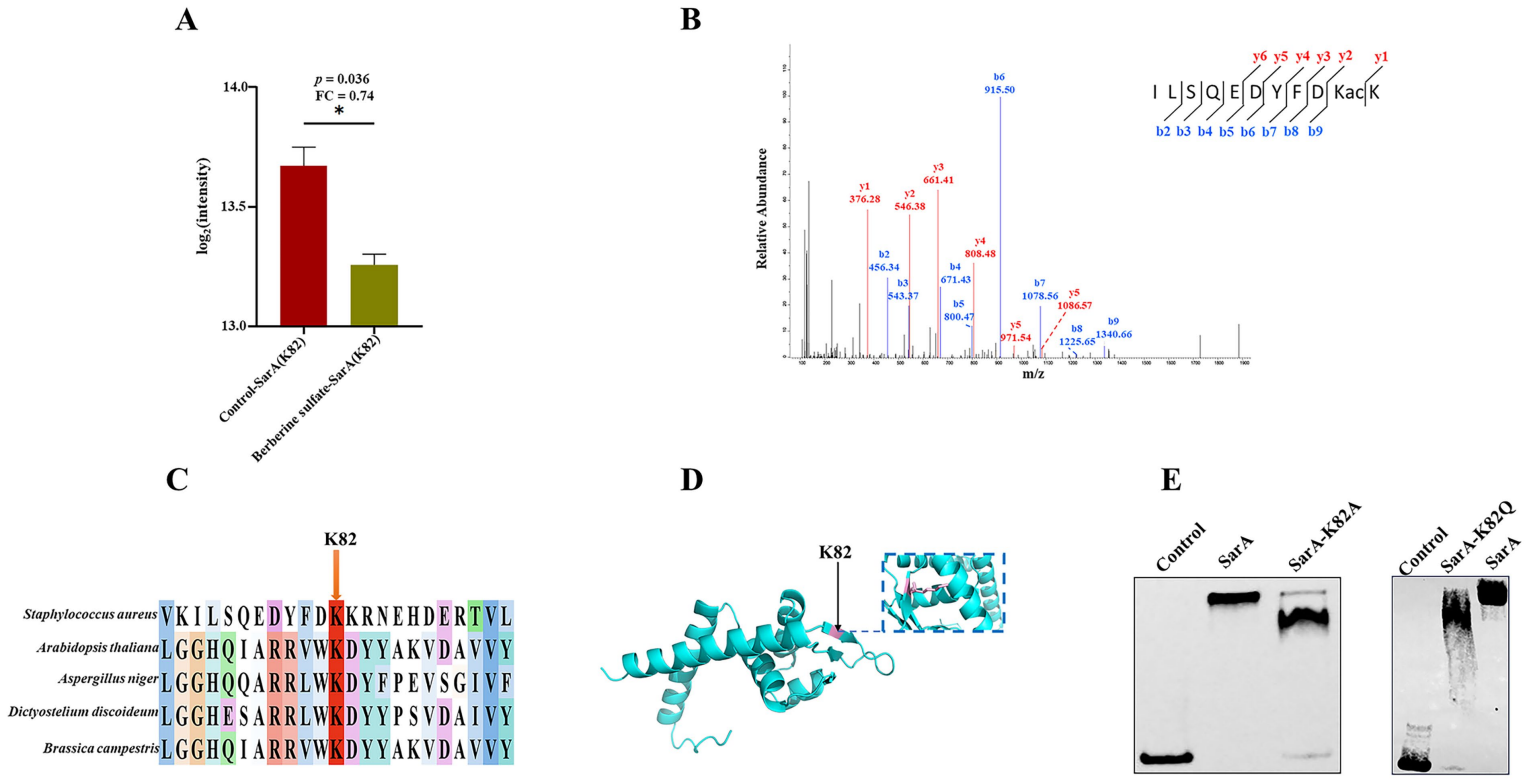
Relative activity of SarA at the site-specific level. **(A)** Bar chart illustrating the differences in acetylation modification of K82 between the berberine sulfate-treated group and the control group; **(B)** MS/MS spectra of acetylated peptides highlighting SarA K82; **(C)** sequence alignment of the transcriptional regulator SarA across various species; **(D)** visualization of the acetylation site within the crystal structure of the transcriptional regulator SarA; **(E)** an electrophoretic mobility shift assay (EMSA) was conducted to confirm the binding affinity of wild-type SarA and its mutants to DNA.

The expression of virulence factors in *S. aureus* is coordinately regulated by global regulatory loci including Agr and SarA (Manna and Cheung, 2006). Our quantitative proteomic analysis demonstrated that treatment with berberine sulfate significantly decreased acetylation at lysine 82 (K82) of SarA (*p* = 0.036, fold change = 0.74) (Figure 7A). The peptide containing this acetylation site was definitively identified via MS2 spectral verification (Figure 7B). Phylogenetic analysis revealed that K82 is highly conserved across multiple species (Figure 7C). The structure of the SarA protein in Figure 7D illustrates the position of the critical residue lysine 82 (K82), which is prominently highlighted. Current evidence indicates that the DNA-binding protein SarA directly regulates the transcription of both core quorum-sensing genes, particularly *agr*, as well as downstream effectors such as *hla* and *spa* (Cheung et al., 2008a). Transcriptomic studies suggest that SarA governs the expression of over 120 genes through both direct and indirect mechanisms (Dunman et al., 2001), with its expression being tightly dependent on the growth phase (Manna and Cheung, 2001). To elucidate the regulatory role of SarA on the *agr* system, we systematically compared the DNA-binding affinity of wild-type SarA and its K82A and K82Q mutants to the *agr* promoter using electrophoretic mobility shift assays (EMSAs). Wild-type SarA formed specific protein-DNA complexes with the *agr* promoter, whereas the binding capacity of both the K82A and K82Q mutants was significantly reduced (Figure 7E). These results suggest that acetylation at K82 modulates SarA’s DNA-binding activity, potentially influencing the expression of MRSA virulence factors.

## 4 Discussion

MRSA represents one of the most formidable multidrug-resistant pathogens encountered in clinical environments, exhibiting inherent resistance to nearly all *β*-lactam antibiotics, including penicillins and cephalosporins (McCarthy et al., 2015; Vestergaard et al., 2019; Nandhini et al., 2022). The ongoing expansion of MRSA resistance profiles has substantially undermined existing therapeutic options, necessitating the development of novel antimicrobial strategies. Natural products have emerged as promising resources for the development of anti-resistance drugs, owing to their distinct antibacterial mechanisms and favorable safety profiles. Berberine sulfate, a pharmacologically active isoquinoline alkaloid and the principal bioactive constituent of the traditional Chinese medicinal herb Coptis chinensis, has demonstrated excellent safety in numerous preclinical studies (Qi et al., 2015; Liu et al., 2019; Li et al., 2023). Its antimicrobial efficacy against a variety of pathogens has been well documented in prior research (Li et al., 2020). Nevertheless, the molecular mechanisms underlying its anti-MRSA activity, particularly the modifications in the systemic regulatory network at the proteomic level, remain inadequately characterized.

It is well-known that bacterial resistance mechanisms are highly complex. Although numerous studies have investigated the acquired resistance mechanisms of Gram-negative bacteria at the proteomic level (Li et al., 2018; Li et al., 2019; Chen et al., 2022), only a limited number of studies have focused on the application of proteomics in Gram-positive bacteria. Employing the standard MRSA strain ATCC 33591 as a model organism, we conducted a systematic investigation into the proteomic dynamics and post-translational modification profiles subsequent to berberine sulfate intervention. This was achieved through the integration of quantitative proteomics with antibody-based enrichment methodologies. Our extensive analysis identified a total of 1,685 proteins, among which 111 were significantly upregulated and 144 were significantly downregulated. Functional annotation indicated that the differentially expressed proteins were predominantly localized within the cytoplasm. Furthermore, KEGG pathway enrichment analysis revealed significant downregulation of metabolic pathways, particularly highlighting changes in the regulation of secondary metabolite biosynthesis. Notably, the metabolic inhibition phenomenon observed in this study aligns with previous research, which has documented a tendency for central carbon metabolism and energy metabolism processes to be attenuated in various drug-resistant bacteria (Cheng et al., 2018; Liu et al., 2021). Key enzymes in these pathways, such as those involved in glycolysis and the TCA cycle, are essential for energy metabolism, responsible for generating NADH and ultimately driving ATP synthesis (Fernie et al., 2004). In this study, we similarly detected significant downregulation of multiple proteins involved in energy metabolism, particularly within the glycolytic pathway (Figure 4A). These results suggest that MRSA may actively reduce its energy metabolism level as a survival strategy to adapt to berberine sulfate stress. A comprehensive analysis of biological processes demonstrated that berberine sulfate treatment specifically activated critical metabolic pathways, such as organic substance metabolism, while concurrently inhibiting pathways associated with small molecule metabolism in MRSA. To elucidate the functional relationships among these differentially expressed proteins, a protein–protein interaction network was constructed utilizing the STRING database. This systematic approach uncovered a dual antimicrobial mechanism of berberine sulfate: (1) the upregulation of ribosomal protein clusters, potentially inducing aberrant translational stress, and (2) the downregulation of key enzymes involved in purine metabolism, leading to impaired nucleotide biosynthesis. This multi-target synergistic intervention pattern provides novel mechanistic insights into the broad-spectrum antibacterial activity of berberine sulfate.

We performed an acetylome enrichment analysis on MRSA samples treated with berberine sulfate. Our findings revealed 715 acetylated sites, among which 25 exhibited increased acetylation and 13 showed decreased acetylation. Previous studies have demonstrated that berberine exerts its antibacterial effects by modulating bacterial cell wall hydrolysis, leukocidin expression, and other pathogenic factors (Lee et al., 2003; Zheng et al., 2023; Zhou et al., 2023). Our analysis of annotated keywords revealed that, regardless of whether they were upregulated or downregulated, acetylated sites within the cytoplasm were significantly enriched, suggesting that berberine sulfate may exert its regulatory role by modulating lysine acetylation in the cytoplasm. Furthermore, KEGG pathway analysis demonstrated that the downregulated acetylation sites were particularly enriched in the arginine biosynthesis and carbon metabolism pathways. Notably, arginine metabolism has been confirmed as a central hub regulating antibiotic tolerance in *S. aureus*—disruption of its biosynthetic pathway can induce a tolerant phenotype by inhibiting protein synthesis (Freiberg et al., 2023). Unlike the known mechanism of substrate depletion, this study revealed that berberine sulfate may intervene at a novel level through post-translational modification: specifically, by downregulating the acetylation of arginine biosynthetic enzymes to directly disrupt the function of this pathway. Biological pathway analysis indicated that the differentially acetylated sites were predominantly associated with the following biological processes: macromolecule biosynthetic processes, carboxylic acid metabolic processes, and cellular macromolecule biosynthetic processes. The observed alterations in acetylation modification levels demonstrated a remarkable consistency with the metabolic pathway disruptions identified in prior proteomic studies. This observation implies that berberine sulfate may specifically perturb the metabolic network of MRSA through a dual mechanism involving both protein expression regulation and post-translational modification. In particular, the altered acetylation states of key enzymes within the arginine biosynthesis pathway could directly influence the equilibrium of bacterial nitrogen metabolism, providing novel molecular insights into its antibacterial effects. To facilitate a comprehensive analysis of the functional relationships among differentially acetylated proteins, we constructed a protein–protein interaction network using the STRING database. This analysis identified the highest-scoring subnetwork (score: 8.50), which consisted of acetylated proteins significantly enriched in cytoplasmic functions. This finding corroborates the results of the annotated keywords functional annotation, suggesting that berberine sulfate may affect pertinent biological processes by modulating the acetylation levels of cytoplasmic proteins.

The SarA protein serves as a global regulatory factor in *S. aureus*, orchestrating virulence through diverse mechanisms (Chien et al., 1998; Dunman et al., 2001; Cheung et al., 2008b). Specifically, *sarA* not only enhances biofilm formation but also suppresses the production of proteases and nucleases, thereby maintaining bacterial pathogenicity (Abdelhady et al., 2014). As a significant repressor of protease synthesis, *sarA* promotes the accumulation of virulence-associated proteins and substantially influences various phenotypes, including biofilm development (Beenken et al., 2003; Zielinska et al., 2012; Loughran et al., 2014). Although the biological functions of SarA have been well recognized (Cheung et al., 2008a), whether its activity is finely regulated by post-translational modifications, particularly acetylation, remains unclear. Notably, our quantitative proteomic analysis identified a novel acetylation modification at lysine 82 (K82) of SarA, exhibiting statistically significant changes. Phylogenetic analyses further revealed that this locus is highly conserved across multiple species, suggesting its critical role in SarA function. To confirm that the K82 site of the SarA protein is the key functional site, we compared the binding properties of wild-type SarA with those of the K82A point mutant to the *agr* promoter region using the EMSA experimental system. The experimental results demonstrated that upon incubation of wild-type SarA with the *agr* promoter probe, a distinct migration lag band was observed, accompanied by the complete disappearance of the free probe signal. This observation suggests that the wild-type protein possesses an exceptionally high binding affinity for the target DNA sequence, which is consistent with previous research findings (Reyes et al., 2011). Compared to the wild-type strain, both the K82A and K82Q mutants exhibited significantly attenuated DNA-binding capacity, manifested by accelerated migration of the protein-DNA complex bands, reduced complex stability, and a marked increase in free probe signal within the reaction system. These findings suggest that the charge characteristics and structural integrity of the K82 residue are crucial for its DNA-binding function. The acetylation modification at the K82 site may modulate its interaction with target gene promoters by altering the side-chain charge state and subsequently inducing conformational changes in the DNA-binding domain of SarA protein. Considering that the *agr* system functions as a central hub for population sensing and virulence regulation in *S. aureus*, the acetylation of SarA at K82 may indirectly disturb the expression of virulence factors by affecting the transcriptional regulation of *agr* and, consequently, the expression of virulence factors.

This study employs an integrative approach combining proteomics and molecular biology to systematically characterize protein dynamics in MRSA following berberine sulfate treatment. Notably, it is the first to identify acetylation at K82 of the global regulator SarA and to elucidate its regulatory function in DNA-binding activity. Nonetheless, several limitations must be acknowledged. Firstly, the absence of chemical proteomics analysis precludes the identification of direct binding targets of berberine sulfate, thereby constraining our understanding of how target engagement affects expression profiles and post-translational modification patterns. Secondly, the lack of metabolomic analysis impedes the exploration of potential relationships between changes in acetylation and its key regulatory metabolites (such as NAD+, NAM, acetate, and acetyl-CoA), limiting a comprehensive interpretation of the causal relationship between berberine sulfate-induced acetylation changes and antibacterial effects. Finally, the exclusive focus on acetylation, while neglecting other critical post-translational modifications (e.g., phosphorylation, succinylation), prompts critical inquiries regarding potential cross-regulatory mechanisms. Future investigations should utilize chemical biology techniques (e.g., photoaffinity labeling, drug affinity responsive target stability) to identify direct molecular targets. Additionally, the integration of metabolomics is essential for analyzing substantial changes in metabolic pathways. Implementing multi-omics strategies will facilitate a systematic exploration of the interactions among various types of modifications. These approaches are anticipated to yield deeper mechanistic insights into the role of acetylation in regulating virulence networks and providing a more comprehensive understanding of *S. aureus* pathogenesis.

## 5 Conclusion

This study provides a comprehensive elucidation of the antibacterial mechanism of berberine sulfate against MRSA. Through quantitative proteomic analysis, it was determined that berberine sulfate treatment significantly alters the protein expression profile in MRSA, notably identifying an acetylation modification at lysine 82 (K82) of the global regulator SarA. Further mechanistic investigations revealed that this post-translational modification influences SarA’s binding affinity to the *agr* promoter region, thereby modulating the expression of virulence factors. These findings offer a substantial theoretical foundation and identify a potential therapeutic target for the development of novel anti-MRSA agents that target bacterial acetylation enzymes or substrates. Future research will aim to optimize the antibacterial activity of berberine sulfate based on these insights to develop more effective therapeutic strategies against drug-resistant bacterial infections.

## Data Availability

The datasets presented in this study can be found in online repositories. The names of the repository/repositories and accession number(s) can be found in the article/Supplementary material.
