# Supplementary material for: Proteomics and acetyl-proteomics reveal the antibacterial mechanism of berberine sulfate against methicillin-resistant Staphylococcus aureus
Source: Front Microbiol. 2026 Jan 14;16:1732962. doi: 10.3389/fmicb.2025.1732962 (PMC12847299; doi:10.3389/fmicb.2025.1732962)
Supplement: Supplementary file 1 [file Supplementary_file_1.docx]

**Supporting Information**

**Proteomics and Acetyl-proteomics reveal the antibacterial mechanism of berberine sulfate against methicillin-resistant *Staphylococcus aureus***

Zunli Hu^1,2#^, Huiling Liu^1,2#^, Mengying Chen^3#^, Jiafu Zhou^2,4^, Yunxu Bian^2^, Chuyao Lin^2,4^, Shuming Liu^2,4^, Yewen Sun ^2*^, Minjia Tan^1,2,3,4,5*^, Jun-Yu Xu^1,2,3,4,5*^.

**Affiliations:**

^1^Guangzhou University of Chinese Medicine, Guangzhou, 510006, Guangdong, China

^2^Zhongshan Institute for Drug Discovery, Shanghai Institute of Materia Medica, Chinese Academy of Sciences, Guangdong, China

^3^School of Chinese Materia Medica, Nanjing University of Chinese Medicine, Nanjing 210023, Jiangsu, China

^4^School of Pharmaceutical Sciences, Southern Medical University, Guangzhou, 510515, China

^5^State Key Laboratory of Drug Research, Shanghai Institute of Materia Medica, Chinese Academy of Sciences, Shanghai, 201203, China

**^#^** These authors contributed equally.

*** Correspondence:**Jun-Yu Xu, Email: [jyxu@simm.ac.cn](mailto:jyxu@simm.ac.cn)

Minjia Tan, Email: [mjtan@simm.ac.cn](mailto:mjtan@simm.ac.cn)

Yewen Sun, Email: [sunyewen0358@zidd.ac.cn](mailto:sunyewen0358@zidd.ac.cn)

**Supplementary Figures**


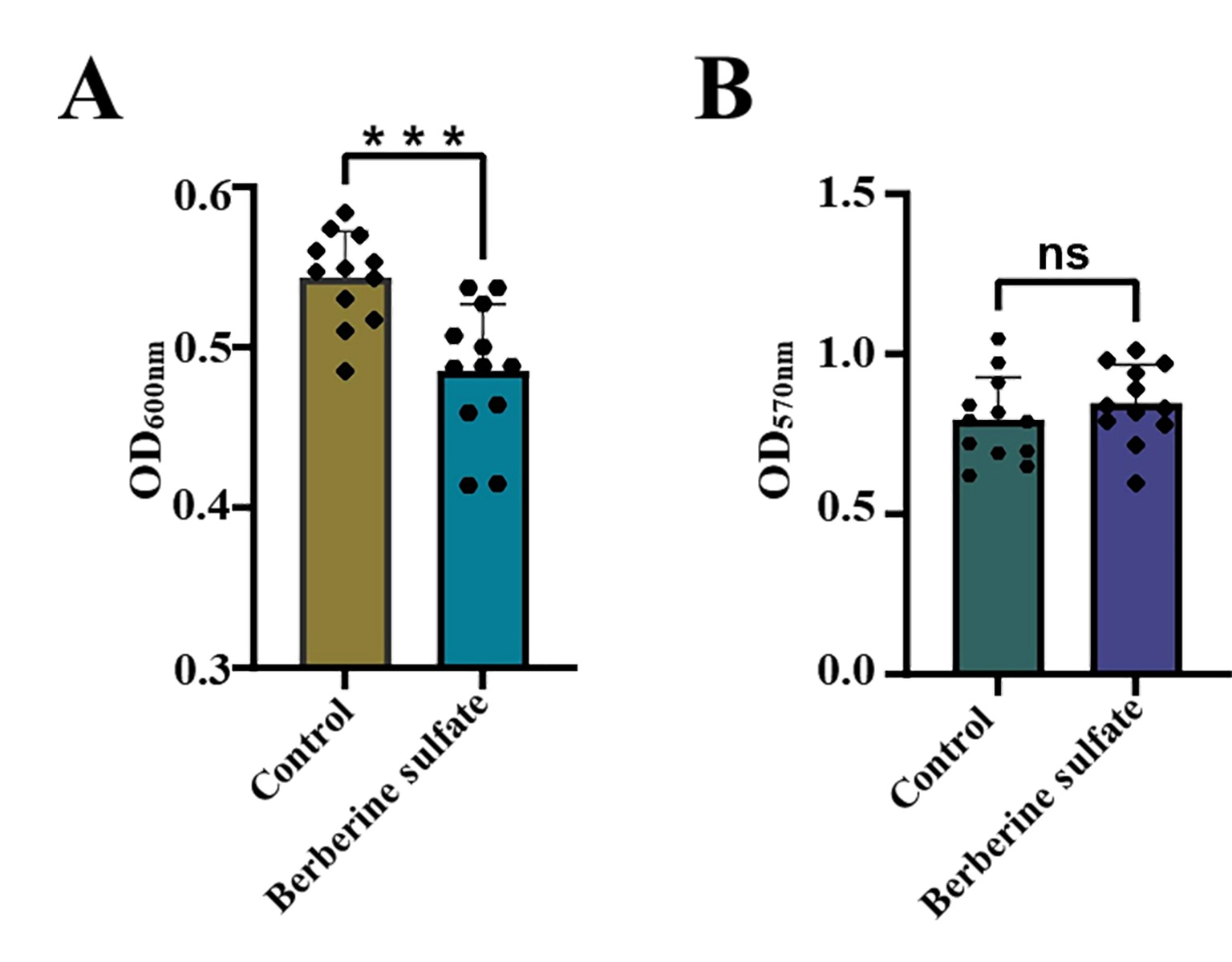


**Figure S1.** Berberine sulfate (50 µM) differentially impacts MRSA growth and biofilm formation. (A) Effect of 24-hour 50 µM berberine sulfate treatment on MRSA planktonic growth (OD600nm). (B) Impact of 24-hour treatment with 50 µM berberine sulfate on MRSA biofilm formation.
